# Myofibroblastoma of the breast in a 5-year-old girl: A case report

**DOI:** 10.1097/MD.0000000000043777

**Published:** 2025-08-08

**Authors:** Youn Joo Jung, Ayoung Kang, Miri Ryu, Soo-Hong Kim, Joon Young Park

**Affiliations:** a Department of Surgery, Pusan National University Yangsan Hospital, Yangsan, Korea; b School of Medicine, Pusan National University, Busan, Korea; c Division of Pediatric Surgery, Pusan National University Children’s Hospital, Yangsan, Korea; d Research Institute for Convergence of Biomedical Science and Technology, Pusan National University Yangsan Hospital, Yangsan, Korea; e Department of Pathology, Pusan National University Yangsan Hospital, Yangsan, Korea.

**Keywords:** breast, case report, myofibroblastoma, pediatrics

## Abstract

**Rationale:**

Myofibroblastoma is a rare, benign spindle cell tumor originating from the breast stroma. Although it predominantly affects older men and occasionally postmenopausal women, its occurrence in children is exceedingly rare.

**Patient concerns:**

A 5-year-old girl presented with a palpable mass on the lateral aspect of her left breast.

**Diagnoses:**

Serial ultrasound examinations were conducted every 6 months for 1 year because the lesion was initially suspected to be a lipoma or an enlarged lymph node.

**Interventions:**

Owing to progressive enlargement of the mass to 3 cm, surgical excision was performed. A well-defined yellow–white solid mass located in the subcutaneous layer was completely removed.

**Outcomes:**

The histopathological and immunohistochemical findings confirmed the diagnosis of a myofibroblastoma. The postoperative course was uneventful, and no recurrence has been reported to date.

**Lessons:**

Although extremely rare, myofibroblastoma should be considered in the differential diagnosis of enlarged breast masses in children. Complete surgical excision is the treatment of choice, and regular follow-up is recommended.

## 1. Introduction

Myofibroblastoma is a rare, benign spindle cell tumor originating in the breast stroma.^[1]^ Although it predominantly occurs in older men and occasionally in postmenopausal women, it is exceedingly rare in children.^[2–4]^ Myofibroblastoma generally presents as a slow-growing solid mass. As its imaging characteristics are nonspecific, accurate diagnosis without histological confirmation is challenging.^[2]^ Herein, we report a case of myofibroblastoma in the breast of a 5-year-old girl, along with a brief review of the literature.

## 2. Case presentation

A 5-year-old girl with no significant medical history presented with a palpable mass on the lateral side of the left breast. Physical examination revealed a well-circumscribed, painless, palpable mass, approximately 1 cm in diameter, in the subcutaneous layer.

**Figure 1.**
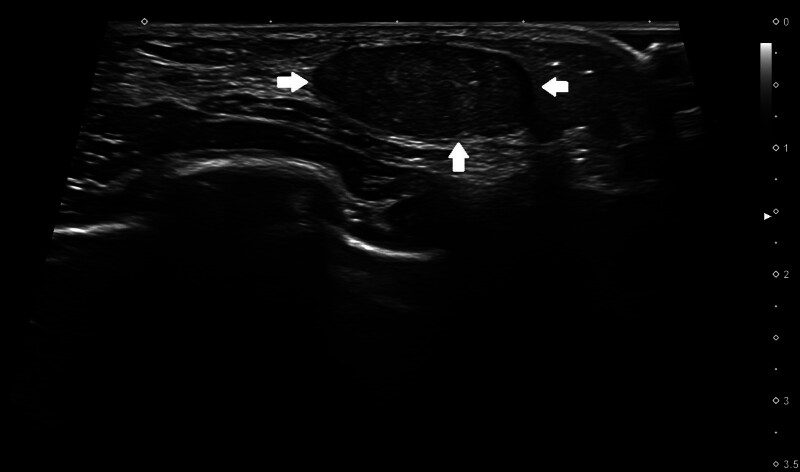
Ultrasound findings showing a well-defined mass measuring 1.7 cm × 1.7 cm × 0.7 cm (arrow), surrounded by a peripheral hypoechoic rim in the left breast.

Ultrasound examination revealed a well-defined, 1.17 cm solid lesion in the subcutaneous layer, filled with echogenic material and surrounded by a peripheral hypoechoic rim. The lesion was initially suspected to be a lipoma or an enlarged lymph node. A follow-up ultrasound performed 6 months later revealed an increased size to 1.7 cm (Fig. 1). A surgical excision was planned if the mass enlarged further.

**Figure 2.**
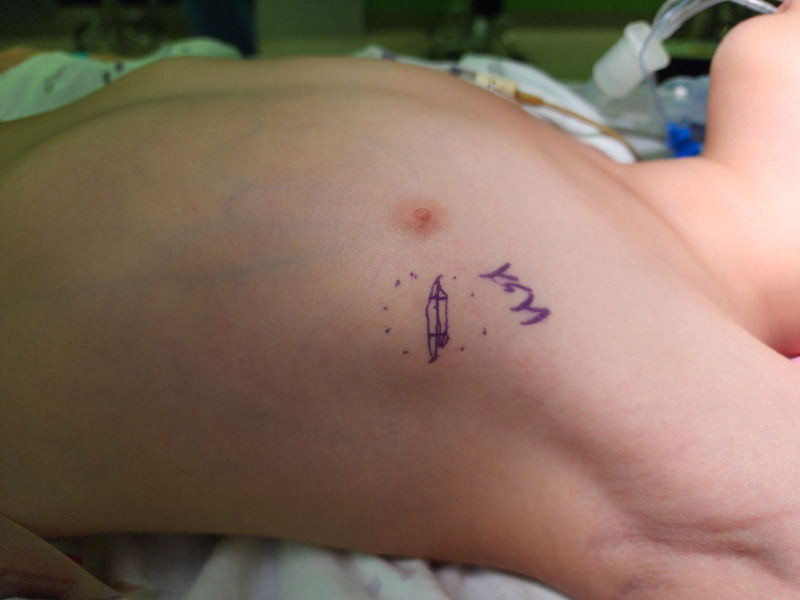
The 5-year-old girl presented with a non-tender, well-circumscribed mass lateral to the left breast.

**Figure 3.**
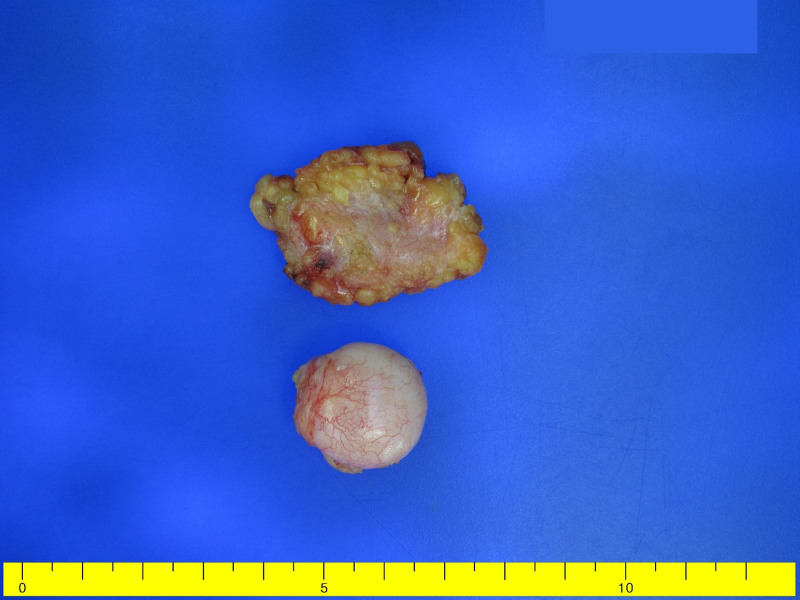
Excised specimen showing a well-circumscribed, yellow-white, solid mass measuring 3.8 cm × 2.5 cm × 0.4 cm.

**Figure 4.**
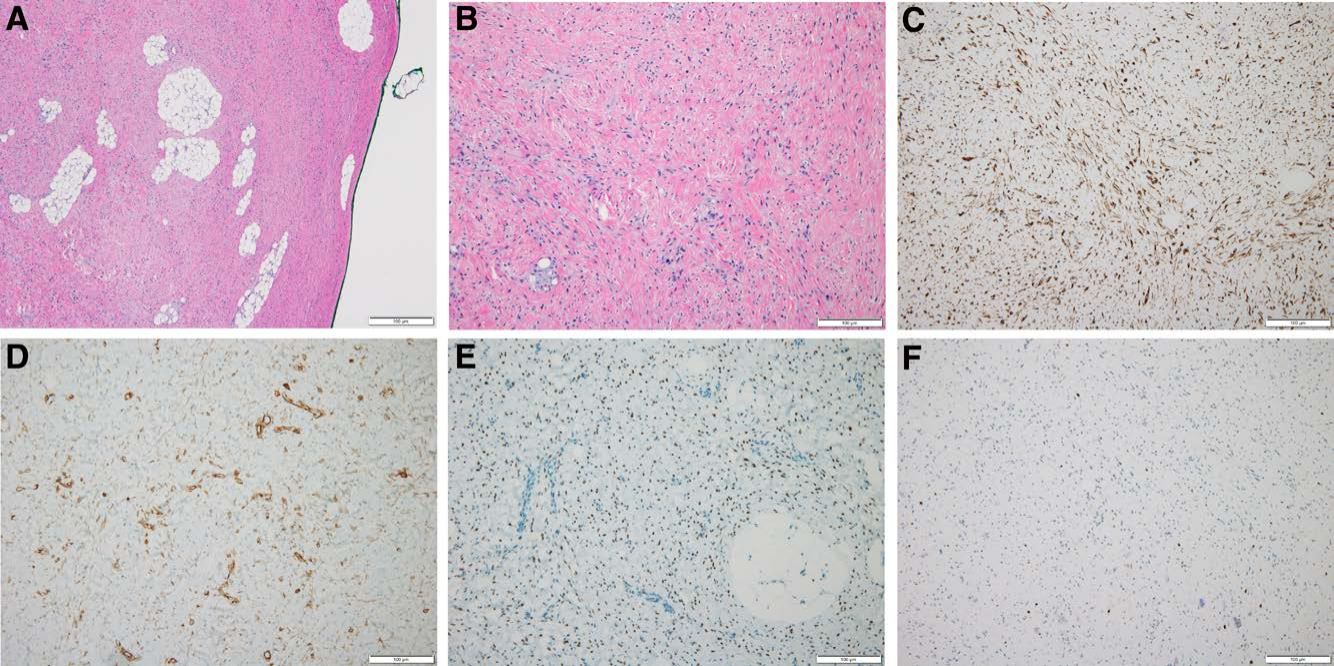
Histological findings confirming the diagnosis of myofibroblastoma. (A) Hematoxylin and eosin (H&E, ×40) staining showing that the mass was well circumscribed without capsule and contained some fat components. (B) H&E (×100) staining showing that the tumor was composed of proliferating oval to spindle cells without a pattern and eosinophilic collagenous stroma. (C) Desmin (×100) staining demonstrating diffuse positivity. (D) Caldesmon (×100) staining demonstrating focal positivity. (E) Estrogen receptor (×100) staining showing focal positivity. (F) The mass showed very low Ki-67 proliferating index (<2%) (×100).

On reassessment 6 months later, the mass had increased to 3 cm, leading to the decision for surgical excision (Fig. 2). Under general anesthesia, an incision was made along the natural skin crease, and the well-defined, yellow-white solid mass found in the subcutaneous layer was completely resected (Fig. 3). Microscopic findings showed that the tumor was well circumscribed and fat components were observed in multifocal areas at low magnification. At high magnification, tumor was composed of oval to spindle cell proliferation without a pattern admixed with eosinophilic collagenous stroma. Immunohistochemical analysis revealed positivity for desmin and focal estrogen receptors as well as caldesmon positivity. The mass was negative for smooth muscle actin, CD34, and S-100 protein. And the mass showed very low Ki-67 proliferating index (<2%). Based on these findings, a diagnosis of myofibroblastoma was made (Fig. 4).

The postoperative course was uneventful, and no recurrence was observed at the last follow-up (1 year postoperatively).

## 3. Discussion

Myofibroblastoma was first described in 1987 by Wargotz et al in a series of 16 cases involving breast masses.^[5]^ However, myofibroblastoma can also occur in organs other than the breast. A higher proportion of cases are being increasingly reported in extra-mammary locations.^[6,7]^ It primarily occurs in men over 40 years of age and in postmenopausal women. In some cases, it has been associated with gynecomastia, suggesting a possible hormonal influence on tumor development.^[2]^ Its association with race, other comorbidities, drugs, other tumors, and growth factors remains unclear.^[8]^ Myofibroblastoma of the breast is extremely rare in children, and cases occurring in girls, such as the present case, are almost nonexistent.^[4]^ To our knowledge, this is one of the very few reported cases of myofibroblastoma in a young girl’s breast.

We observed the mass for 1 year without performing a biopsy, adhering to the principle of “first, do no harm” in the management of breast masses in children. Most pediatric breast masses are benign. A thorough physical examination is essential; if imaging is warranted, ultrasonography is the preferred modality. As normally developing breast tissue at various Tanner stages may present as palpable masses, careful clinical evaluation is crucial. Unnecessary biopsies should be avoided to prevent potential injury to the developing breast.^[9]^

Histological examination is essential for establishing a definitive diagnosis. In this case, given the lesion’s continuous growth over a 1-year period and the initial differential considerations of lipoma and enlarged lymph nodes, an excisional biopsy was performed. Although imaging findings are typically nonspecific, core needle biopsy is recommended for tissue diagnosis in adult patients. Fine-needle biopsy often reveals only atypical whorls of spindle cells, making an accurate diagnosis challenging.^[2]^

Myofibroblastomas are thought to originate from mammary stromal fibroblasts, and are composed of fibroblasts, myofibroblasts, and a variable number of adipocytes. Grossly, it typically presents as a yellow-to-tan, well-circumscribed, round, oval, or lobulated mass with a firm and rubbery consistency.^[2,10]^ Microscopically, it is characterized by randomly arranged, bland-appearing spindle cells and interspersed adipocytes within collagenous and myxoid stroma containing hyalinized collagen fibers, all surrounded by a pseudo-capsule. Mitotic figures, cytological atypia, and necrosis are generally absent.^[2,3,10]^

Immunohistochemical staining plays a crucial role in the diagnosis of myofibroblastomas. In accordance with myofibroblastic differentiation of the tumor, positive staining for smooth muscle actin, desmin, vimentin, and CD34 is typically observed. In contrast, caldesmon positivity may suggest differentiation toward a leiomyomatous or smooth muscle lineage.^[1,8]^ In most cases, positive staining for estrogen, progesterone, and androgen receptors and variable staining for CD99 and Bcl-2 is observed. Most cases also demonstrate positive staining for estrogen, progesterone, and androgen receptors, along with variable expression of CD99 and Bcl-2.^[1]^ The tumor is usually S-100 protein negative, which is typically expressed in schwannomas and other spindle cell tumors.^[4,8]^

The treatment of choice is a complete surgical resection. Although data on spontaneous regression are limited, the lesions do not appear to resolve independently. Recurrence following complete resection is extremely rare, and there have been no reports of malignant transformation or metastasis in English literature. However, regular and long-term follow-up is still recommended.^[2–4,8]^

## 4. Conclusions

In conclusion, although extremely rare, myofibroblastoma should be considered in the differential diagnosis of enlarged breast masses in children. Complete surgical excision is the treatment of choice, and regular follow-up is recommended.

## Author contributions

**Conceptualization:** Soo-Hong Kim.

**Supervision:** Soo-Hong Kim.

**Writing – original draft:** Youn Joo Jung, Ayoung Kang, Miri Ryu.

**Writing – review & editing:** Soo-Hong Kim, Joon Young Park.
